# A New Resveratrol Trimer from the Roots and Stems of *Vitis wenchowensis*

**DOI:** 10.3390/molecules18077486

**Published:** 2013-06-27

**Authors:** Binbin Gu, Yaru Xu, Shan He

**Affiliations:** School of Marine Sciences, Ningbo University, Ningbo 315211, China; E-Mails: gubin21314@gmail.com (B.G.); XuYaRulovelife@163.com (Y.X.)

**Keywords:** *Vitis wenchowensis*, resveratrol oligomer, wenchowenol, Vitaceae, antioxidant, DPPH

## Abstract

Phytochemical constituents of *Vitis wenchowensis* were investigated for the first time. A new resveratrol trimer, wenchowenol (**1**), was isolated from the roots and stems of *Vitis wenchowensis* along with four known stilbenoids **2–5**. The structure and relative configuration of **1** were established on the basis of spectral evidence, especially HMBC and NOESY experiments. It showed potent antioxidant activity against DPPH (1,1-diphenyl-2-picrylhydrazyl) radical.

## 1. Introduction

Resveratrol, a phytoalexin isolated from diverse plant families, has become one of the most extensively studied natural products, since it was involved in the health benefits associated with a moderate consumption of red wine (the so-called “French paradox”) in the early 1990s [1,2]. Numerous *in vitro* and *in vivo* studies have demonstrated its great potential to prevent or treat a wide variety of diseases, including cardiovascular disease, cancer, and neurodegenerative diseases, as well as to extend lifespan [3].

Nevertheless, in some particular families of plants (e.g., Vitaceae, the grapevine family), a large number of phytoalexins were polymerized as resveratrol oligomers, arising from the oxidative coupling of two to eight units of resveratrol monomer [4]. They have attracted considerable attention not only for their structural diversity and biological activities, but also owing to their vital roles in plant defense mechanisms [5].

Our previous phytochemical investigations on Vitaceae plants have led to discovery of novel bioactive resveratrol oligomers [6,7,8]. Our continued research on chemical constituents of the roots and stems of *Vitis wenchowensis* has now resulted in the isolation of a new resveratrol trimer, wenchowenol (**1**) together with four known stilbenoids **2–5**. In this paper, we report the structural characterization of **1** and its antioxidant activity against DPPH (1,1-diphenyl-2-picrylhydrazyl) radicals.

## 2. Results and Discussion

**Figure 1:**
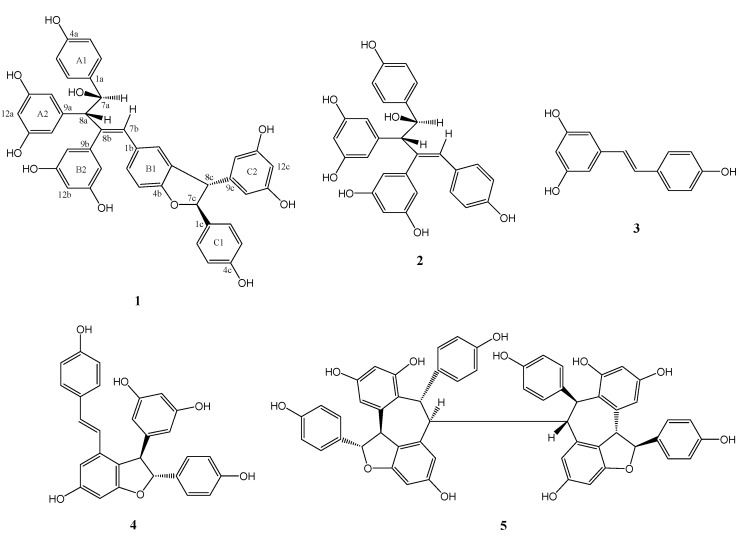
Chemical structures of stilbenoids from *V.* *wenchowensis*.

The roots and stems of *V.* *wenchowensis* were extracted with methanol at room temperature to yield a crude extract. This crude extract was then partitioned between ethyl acetate and water. The ethyl acetate solubles were separated by silica gel column chromatography (CC), followed by preparative HPLC to afford compounds **1**–**5** (Figure 1).

Wenchowenol (**1**) was obtained as colorless amorphous powder, which was determined to have a molecular formula of C_42_H_34_O_10_ from its HR-ESI-MS, which corresponded to a hydroxylated resveratrol trimer, since the typical formula of resveratrol trimers is C_42_H_32_O_9_. The ^1^H-NMR and ^1^H,^1^H-COSY spectra of **1** showed the presence of two sets of *ortho*-coupled aromatic H-atoms assignable to two 4-hydroxyphenyl groups, three sets of 3,5-dihydroxyphenyl groups, a set of H-atom forming a ABX system on a 1,3,4-trisubstituted benzene ring, an olefinic H-atom, and two sets of mutually coupled methine H-atoms. 

**Figure 2:**
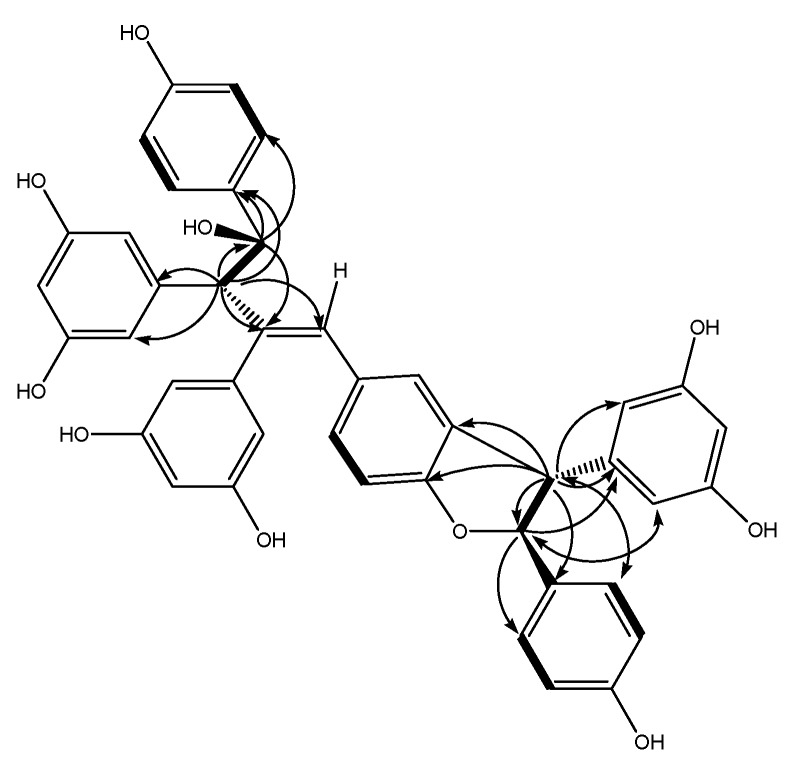
Key HMBC (indicated by arrows from ^1^H to ^13^C), ^1^H–^1^H COSY correlations (indicated by bold lines), and NOESY correlations (indicated by double-headed arrows between two protons) for **1**.

A comparison between the NMR data of **1** and those of the co-occurring amurensin A (**2**), which has been previously isolated from *V. amurensis* [9], revealed that **1** contained **2** as partial structure (resveratrol units A and B). Compound **1** showed signals (Table 1, signals from 1c to 14c) corresponding to an additional resveratrol unit C, which was confirmed by HMBC data (Figure 2). The HMBCs H-8c/C-3b, C-4b, and C-10c(14c) and H-7c/C-2c(6c) and C-9c indicated that the resveratrol unit C formed a dihydrofuran ring fused with the aromatic ring B1, while the 4-hydroxyphenyl group (ring C1) was attached at C-7c. Thus the structure of **1** was determined as shown in Figure 1. Its ^1^H and ^13^C-NMR data were assigned in Table 1.

**Table 1:** ^1^H and ^13^C-NMR Data of wenchowenol (**1**) in acetone-*d*_6_*^a^*.

position	*δ*_H_ (mult., *J* Hz)	*δ*_C_		position	*δ*_H_ (mult., *J* Hz)	*δ*_C_
1a		135.7		7b	6.73 *^b^*	128.2
2a (6a)	7.03 (2H, *d*, 8.5)	129.5		8b		142.3
3a (5a)	6.56 (2H) *^b^*	115.6		9b		144.8
4a		157.4		10b (14b)	6.01 *^b^*	109.2
7a	5.02 (1H, *d*, 10.4)	76.0		11b (13b)		159.4
8a	3.68 (1H, *d*, 10.5)	64.6		12b	6.12 (1H, *t*, 2.0)	102.3
9a		144.0		1c		132.9
10a (14a)	5.94 (2H, *d*, 2.0)	109.2		2c (6c)	7.10 (2H, *d*, 8.5)	128.6
11a (13a)		158.5		3c (5c)	6.74 (2H, *d*, 8.9)	116.3
12a	5.91 (1H, *t*, 1.9)	101.5		4c		158.8
1b		132.3		7c	5.27 (1H, *d*, 8.2)	94.5
2b	6.86 (1H, *d*, 8.2)	130.6		8c	4.23 (1H, *d*, 8.0)	58.8
3b	6.55 *^b^*	109.3		9c		145.3
4b		159.6		10c (14c)	6.01 *^b^*	107.7
5b		131.0		11c (13c)		159.9
6b	6.76 *^b^*	127.7		12c	6.16 (1H, *t*, 2.5)	102.5

*^a^* ^1^H-NMR spectra were measured at 500 MHz, and ^13^C-NMR spectra were run at 125 MHz; *^b^* Overlapping (in the same column).

In order to clarify the stereochemistry of **1**, a NOESY (Figure 2) experiment was carried out. The NOEs between H-8c and H-2c(6c), H-7c and H-10c(14c) illustrated that H-8c and H-7c should be *trans* oriented. A comparison of NMR data between molecules **1** and **2** [1] suggested that H-8a and H-7a has a *trans* orientation, which was identical with **2**. Thus the stereochemistry was as shown in Figure 1. Compound **1** showed potent scavenging activity against DPPH radical with IC_50_ = 43 μM, which was comparable with resveratrol (IC_50_ = 38 μM). 

**Figure 3:**
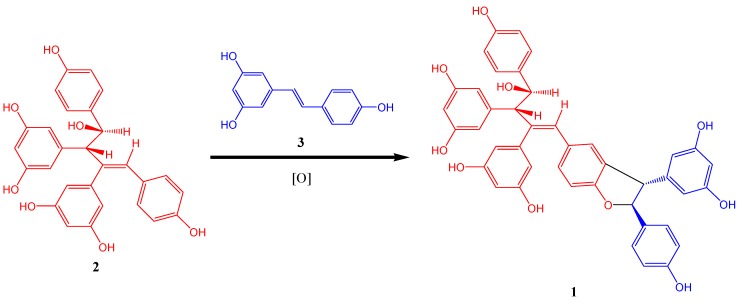
Postulated Biogenetic Pathway of **1**.

Although oligomerization of stilbenes could generate various skeleton types, formation of dihydrofuran ring usually occurs during oxidative coupling [8]. Therefore **1** was probably generated by oxidative coupling between **2** and resveratrol. A proposed biogenetic pathway is shown in Figure 3.

## 3. Experimental

### 3.1. General Methods

^1^H-NMR spectra were recorded at 500 MHz, and ^13^C-NMR spectra were recorded at 125 MHz with tetramethylsilane (TMS) and solvent signals as internal references. HR-ESI-MS data were acquired with a FT-ICR mass spectrometer. IR spectra were measured on a FT-IR spectrometer as KBr pellets. Column chromatography (CC) was carried out on silica gel (200–300 mesh from Qingdao Marine Chemical Co. Ltd., Qingdao, China).

### 3.2. Chemicals

1,1-Diphenyl-2-picrylhydrazyl (DPPH) were purchased from Sigma (St. Louis, MO, USA). Solvents (analytical grade) for extraction and CC were purchased from Huadong Chemicals (Hangzhou, China).

### 3.3. Plant Material

The roots and stems of *V.* *wenchowensis* were collected in June 2007 in Wenzhou, Zhejiang Province, China. The material was identified by Dr. Yunpeng Zhao (College of Life Sciences, Zhejiang University, Hangzhou, China). A voucher specimen (No. P070674) was deposited at the Department of Biology, Zhejiang University, China.

### 3.4. Extraction and Isolation

The plant material (1.8 kg) was extracted three times with MeOH (3 × 20 L) at room temperature. The solvent was evaporated *in vacuo* to produce a concentrated MeOH extract (155 g), which was then diluted with H_2_O (1 L) to give an aqueous solution (1 L). The aqueous solution was extracted with EtOAc three times (3 × 2.0 L). The combined EtOAc layers were condensed *in vacuo* to provide an EtOAc extract (82 g), which was then subjected to silica gel CC (1,000 g, 5 cm diameter column) eluted with light petroleum-EtOAc mixtures (10:1 to 1:10) to yield eight fractions. Fraction 3 (1.3 g) was subjected to preparative HPLC (column YMC-C18, 250 × 20 mm i.d.; solvent MeOH-H_2_O, 40%:60%; flow rate 8 mL/min; detection 280 nm) to afford two pure isolates **3** (*t*_R_ = 28 min, 75 mg), **2** (*t*_R_ = 51 min, 40 mg) and **4** (*t*_R_ = 84 min, 120 mg). Fraction 5 (1.6 g) was separated by preparative HPLC under similar conditions except for the ratio of MeOH: H_2_O (45%:55%) to give compounds **1** (*t*_R_ = 48 min, 24 mg), and **5** (*t*_R_ = 79 min, 150 mg).

Wenchowenol (**1**): colorless amorphous powder; [*α*]^20^_D_ +21 (*c* 0.25, MeOH); UV (MeOH) *λ*_max_ (log *ε*) 228 (4.5), 284 (3.2), 315 (3.2) nm; IR (KBr) *ν*_max_ 3387, 1611, 1516, 1444, 1332, 1236, 1174, 1001, and 834 cm^−1^; ^1^H and ^13^C NMR data, see Table 1; HR-ESI-MS *m*/*z* [M - H]^−^ 697.2078 (calcd for C_42_H_33_O_10_, 697.2074).

Compounds **2–5** were determined as amurensin A [9], resveratrol [10], *ε*-viniferin [11], and hopeaphenol [12], respectively, according to the spectroscopic data in the literature.

### 3.5. Determination of Antioxidant Activities

The antioxidant activities of **1** were determined by the DPPH assay as previously described [8]. Briefly, the reaction mixture containing sample solution (20 μL) and DPPH (180 μL, 150 μM) in ethanol was placed in a 96-well microplate and incubated at 37 °C for 30 min. The absorbance was measured at 517 nm by a microplate reader. IC_50_ value represents the concentration of a compound to scavenge 50% of DPPH radicals. Resveratrol was used as positive control.

## 4. Conclusions

In present study, phytochemical investigation on the roots and stems of *Vitis wenchowensis* has led to the isolation of a new resveratrol trimer, named wenchowenol (**1**), which is probably the oxidative coupling product of amurensin A and resveratrol. DPPH assay demonstrated that **1** is a potent antioxidant comparable to the famous red wine polyphenol resveratrol.

## Supplementary Materials

Supplementary materials can be accessed at: http://www.mdpi.com/1420-3049/18/7/7486/s1.
