# Supplementary material for: A New Resveratrol Trimer from the Roots and Stems of Vitis wenchowensis
Source: Molecules. 2013 Jun 27;18(7):7486–91. doi: 10.3390/molecules18077486 (PMC6270250; doi:10.3390/molecules18077486)

Supplementary Materials

General Experimental Methods

^{1}H NMR spectra were recorded at 500 MHz, and ^{13}C NMR spectra were recorded at 125 MHz with tetramethylsilane (TMS) and solvent signals as internal references. HR-ESI-MS data were acquired with a FT-ICR mass spectrometer. IR spectra were measured on a FT-IR spectrometer as KBr pellets. Column chromatography (CC) was carried out on silica gel (200–300 mesh from Qingdao Marine Chemical Co. Ltd., China).

Chemicals

1,1-diphenyl-2-picrylhydrazyl (DPPH) were purchased from Sigma (St. Louis, MO, USA). Solvents (analytical grade) for extraction and CC were purchased from Huadong Chemicals (Hangzhou, China).

Figure S1. ^{1}H NMR spectrum of **1**.

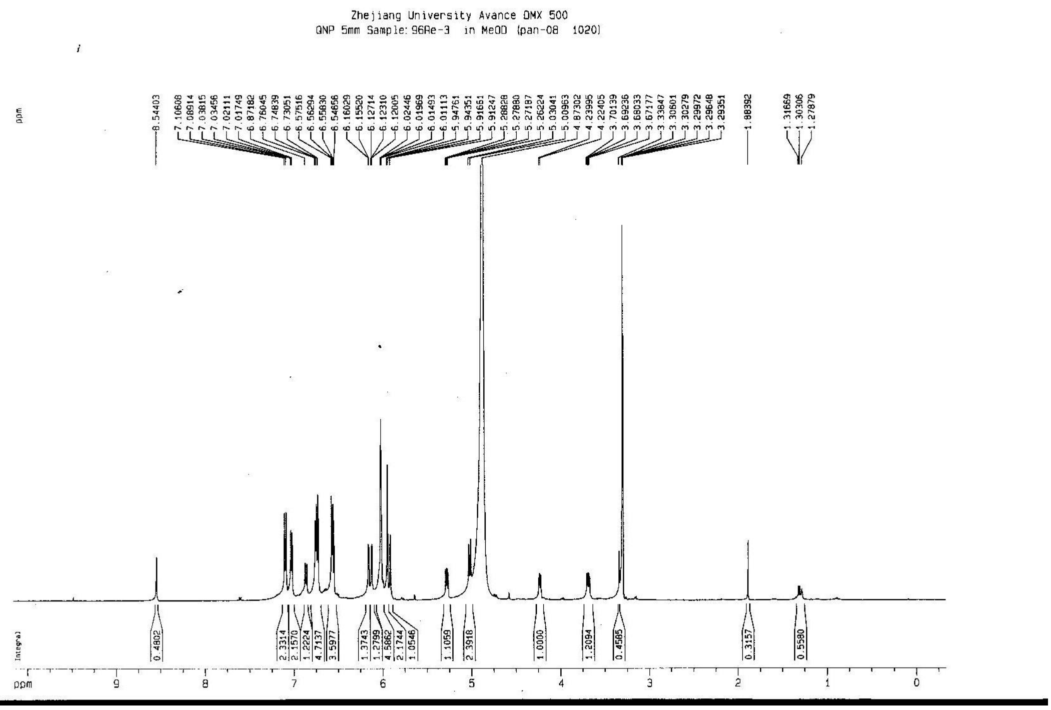

Figure S2. ^{13}C NMR spectrum of **1**.

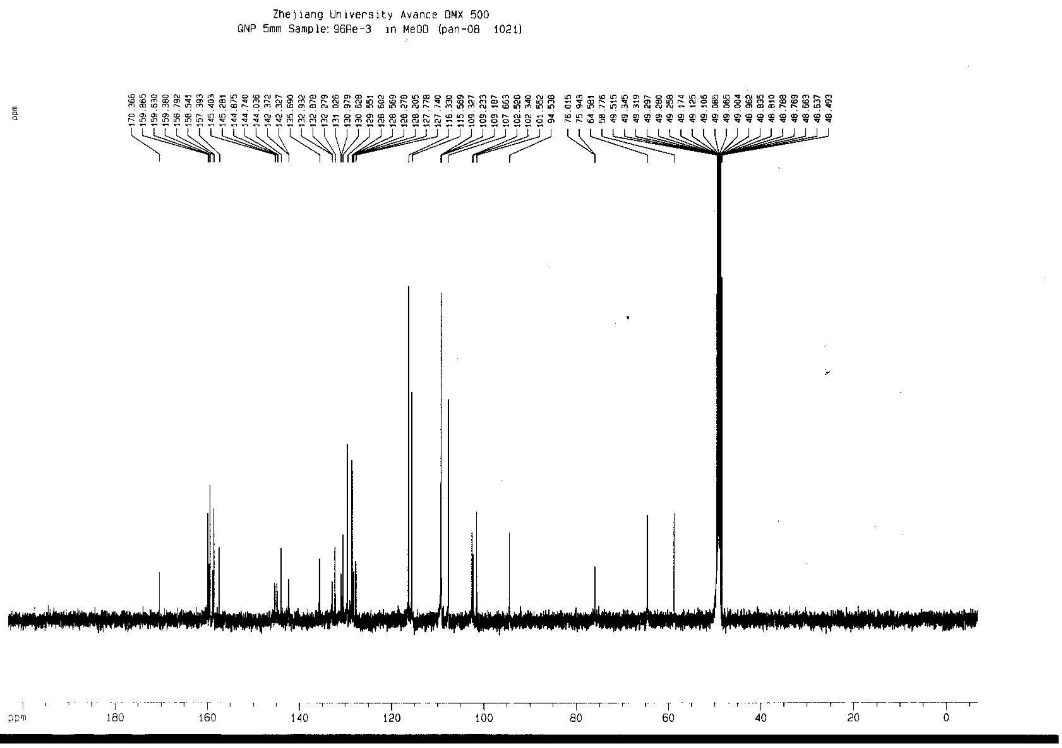

Figure S3. DEPT 135° spectrum of **1**.

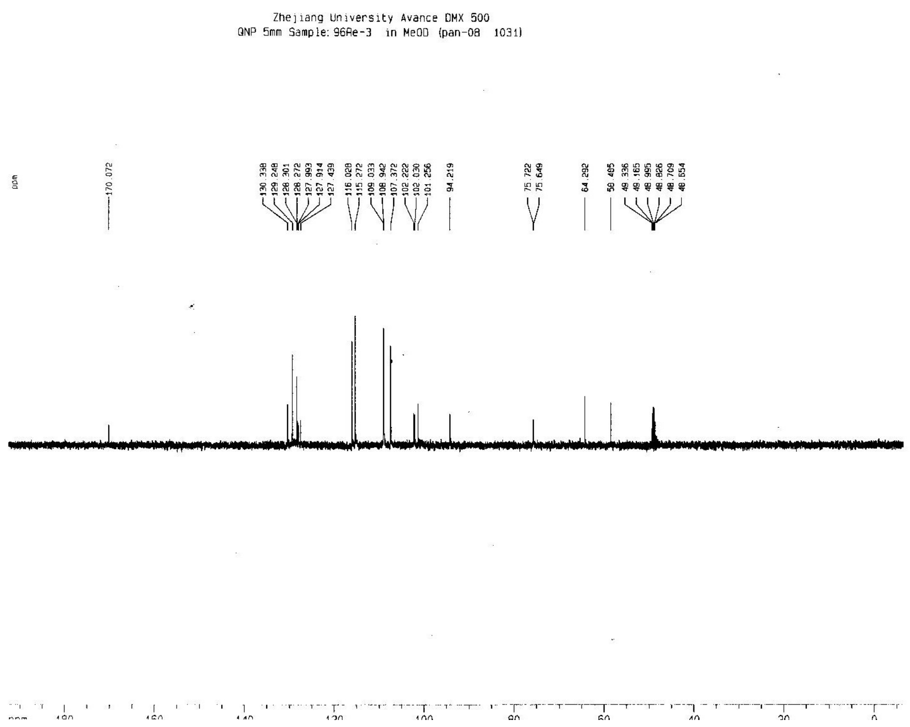

Figure S4. ^{1}H-^{1}H COSY spectrum of **1**.

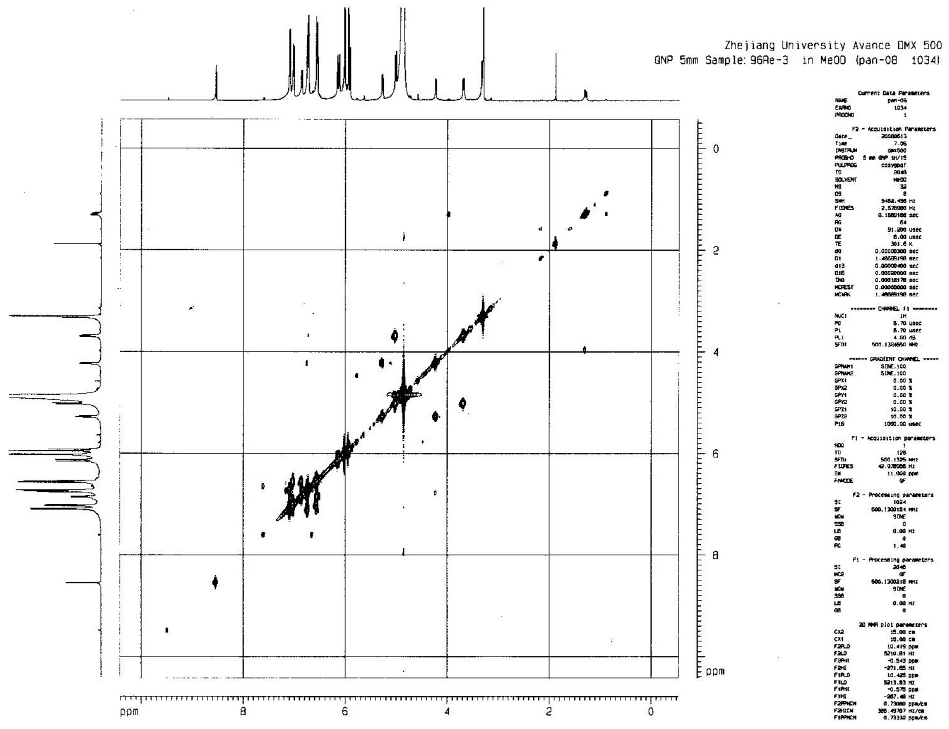

Figure S5. HMQC spectrum of **1**.

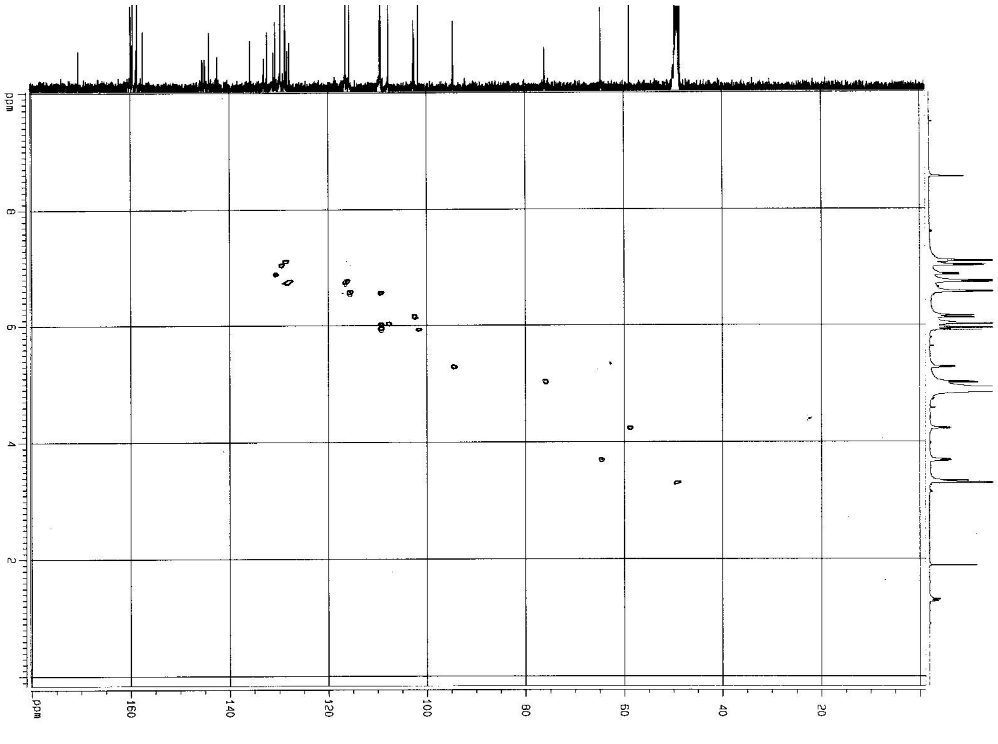

Figure S6. HMBC spectrum of **1**.

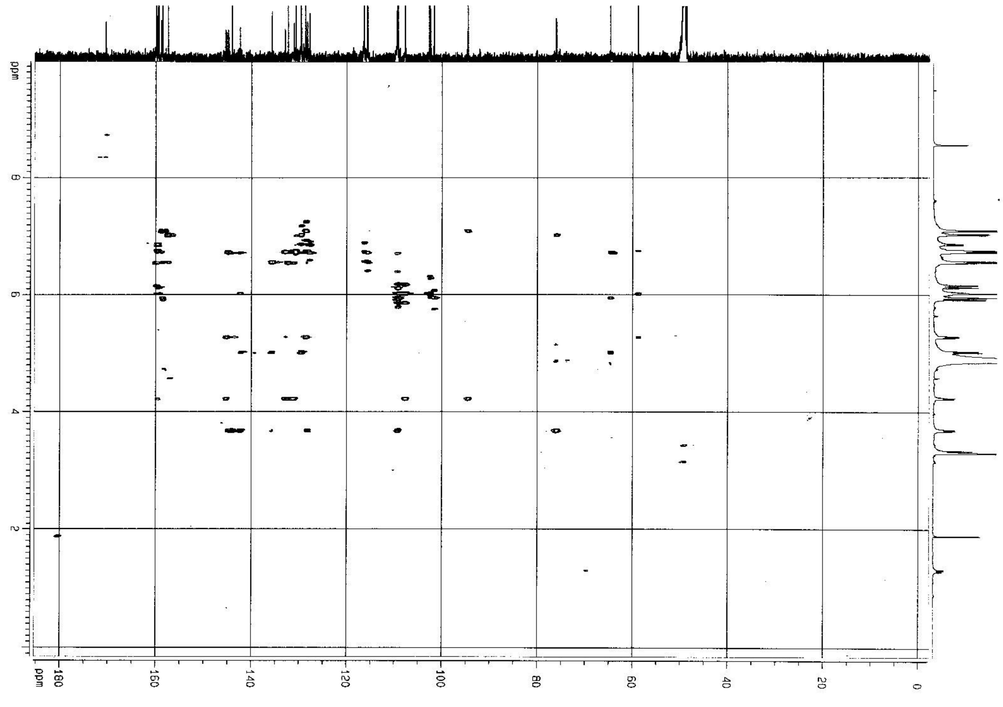

Figure S7. NOESY spectrum of **1**.

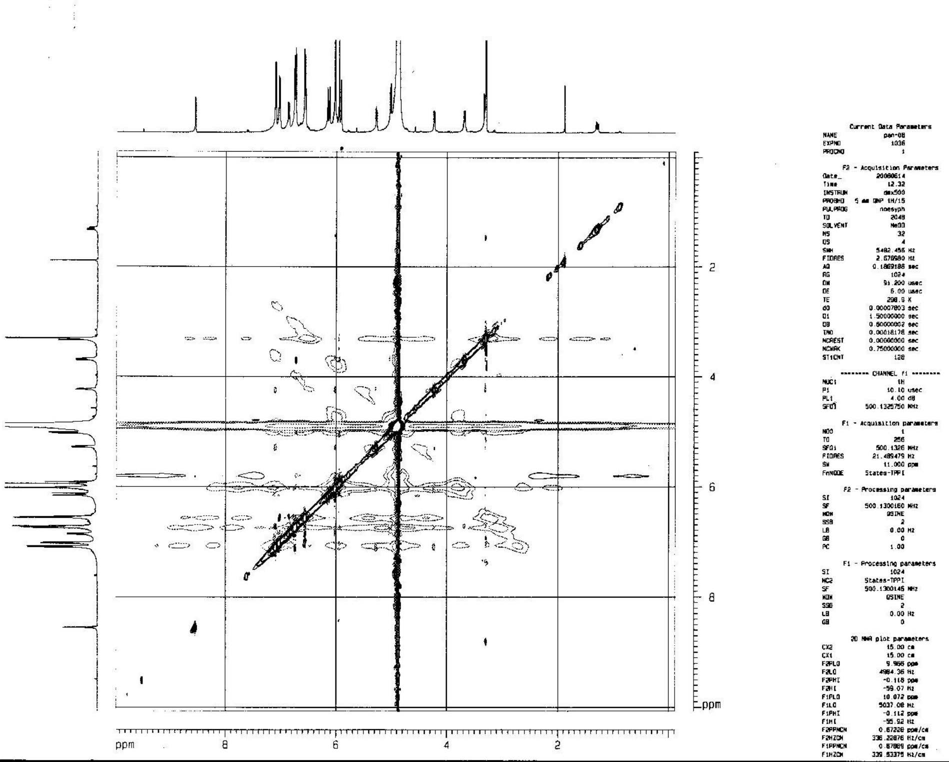

Figure S8. HRMS spectrum of **1.**

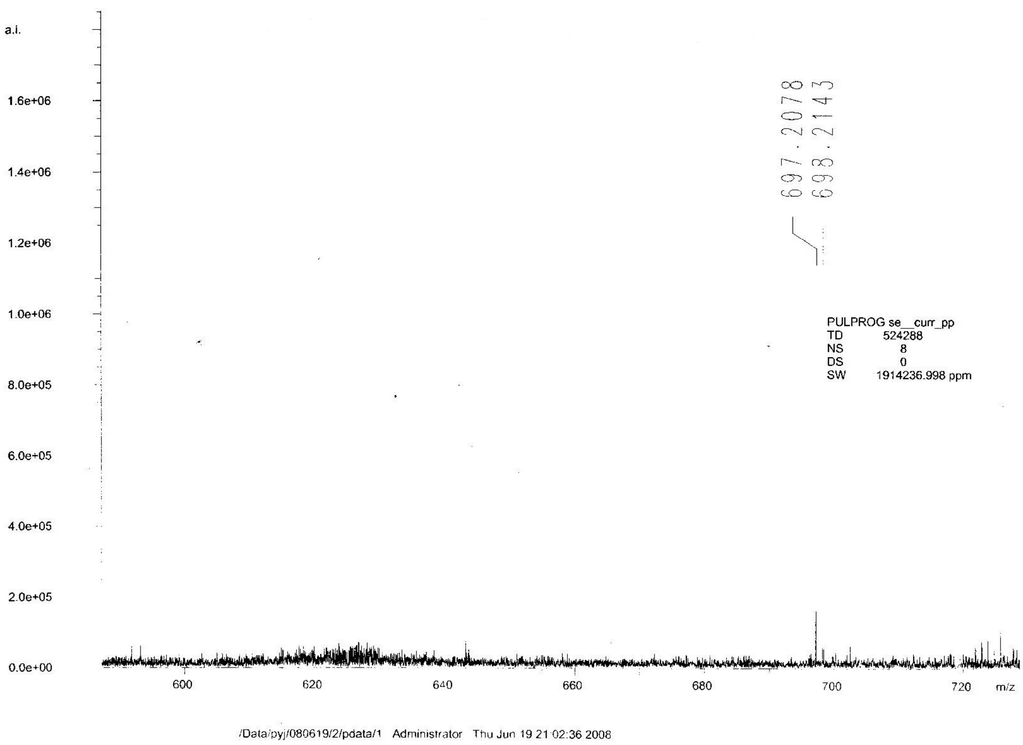

Supplement: Supplementary file 1 [file molecules-18-07486-s001.pdf]
